# Nutritional quality and sensory acceptability of complementary food blended from maize (*Zea mays*), roasted pea (*Pisum sativum*), and malted barley (*Hordium vulgare*)

**DOI:** 10.1002/fsn3.376

**Published:** 2016-05-06

**Authors:** Obse Fikiru, Geremew Bultosa, Sirawdink Fikreyesus Forsido, Mathewos Temesgen

**Affiliations:** ^1^ Department of Food Science and Postharvest TechnologyAmbo UniversityAmboEthiopia; ^2^ Department of Food Science and TechnologyBotswana College of AgriculturePrivate Bag 0027GaboroneBotswana; ^3^ Department of Postharvest ManagementJimma UniversityJimmaEthiopia; ^4^ Department of BiologyAmbo UniversityAmboEthiopia

**Keywords:** Complementary food, maize, malted barley, proximate composition, roasted Pea, sensory quality

## Abstract

The aim of this study was to evaluate the effect of blending ratio of malted barley, maize, and roasted pea flour on complementary food quality and sensory acceptability. D‐ Optimal mixture design was used to generate 14 formulations. Each ingredient had 55–90% maize, 20–35% pea and 4–12% malted barley. Pretreatments like debranning of maize, roasting of pea and dehusking of malted barley were done. The three component‐constrained mixture design was conducted using Design‐Expert^®^ 6 (Stat‐Ease). Ash, protein, fat, fiber, moisture, and carbohydrate contents were found in between range of 1.5–2.5%, 13.0–18.5%, 1.8–2.5%, 3.06–4.45%, 5.0–6.5%, and 68.9–74.1%, respectively. Significant difference (*P* < 0.05) among the treatments was observed for protein, moisture, odor, flavor and sensory overall acceptability. Lack‐of‐fit was significantly different only for fat (*R* ^*2*^ = 0.90). Thus, the model generated can predict all attributes except for fat. The optimum values of high nutrient content and sensory acceptability were observed in the range of 55.0–68.5%, 27.5–35.0%, and 4.0–10.0% for maize, pea, and malted barley respectively.

## Introduction

Complementary foods are any nutrient‐containing foods or liquids other than breast milk given to young children during the period of complementary feeding (6–24 months) (WHO 2001). The growth of an infant in the first 2 years is very rapid and breast feeding alone will not meet the child nutritional requirements. The ability of breast milk to meet the requirements for macronutrients and micronutrients becomes limited with the increasing age of infants (Agostoni et al. 2008). Thus, timely introduction of complementary foods during infancy is necessary for both nutritional and developmental reasons (Agostoni et al. 2008; Kamchan et al. 2004). However, the capacity of a complementary diet to meet the protein‐energy requirements of infants depends on its nutritional quality (Kamchan et al. 2004).

It is well known that high cost of fortified complementary foods in many parts of developing countries is beyond the reach of most families (Amankwah et al. 2009; Muhimbula et al. 2011). Hence, many families depend on inadequately processed and low‐quality traditional complementary foods for their children. That is why protein‐energy malnutrition is a major infant problem in the developing countries (WHO 2001). Therefore, inadequate complementary food is a major cause for the high incidence of child malnutrition, morbidity, and mortality in many developing countries (Krebs and Westcott 2002). To reduce these problems, low‐cost indigenous and unexploited legumes which can be processed and when properly complemented with commonly available carbohydrate sources will provide relatively affordable complementary foods that will help to alleviate protein‐energy malnutrition and improve infants' nutrition (Amankwah et al. 2009; Mbata et al. 2009; Muhimbula et al. 2011). Legumes are known to contain lysine in a quantity that exceeds the requirements for human nutrition, but are with the low content of sulfur‐containing amino acids (Davidson et al. 1980). According to Davidson et al. (1980), cereals also have sulfur amino acids but, deficient in lysine. A mutual complementation of amino acids and consequent improvement in protein quality is therefore achieved when legumes are blended with cereals in the right proportions (Gibson and Hotz 2001).

In Ethiopian, complementary foods given to infants by mothers or caretakers are deficient both in macronutrients (protein, carbohydrates, and fat) and micronutrients (minerals and vitamins) (Zewditu et al. 2001). Therefore, adequate nutrition and health care during the first 2 years of infant life is very essential to prevent this malnutrition (Temesgen 2013). Some researchers tried to improve these problems, but still the protein‐energy‐malnutrition remains a serious problem (Zewditu et al. 2001). Generally, the use of low price and locally available foods formulated in the home has high potential to supplement breast feeding. These are also very important to address the limitation of complementary foods in developing countries (Dewey and Brown 2003; Muhimbula et al. 2011). Therefore, here we aim to evaluate the effect of blending a ratio of maize as a source of starch, roasted pea as a source of protein and malted barley as a source of enzymes and carbohydrates on nutritional and sensory quality of complementary foods was evaluated.

## Materials and Methods

The experiment was conducted at Jimma University, College of Agriculture and Veterinary Medicine (JUCAVM), Postharvest laboratory in 2012 which is located at the south western part of Ethiopia 345 km from Addis Ababa. Samples were collected from three different organizations. Maize (*Zeamays* var., BH660) (50 kg) was taken from Ethiopian seed enterprise Nekemte Branch, Barley (*Hordium vulgare* var., Miscal 21) (20 kg) and peas (*Pisum Sativum* var.,adi) (30 kg) were from Holota Agricultural research center, Ethiopia.

### Sample preparation

Maize, pea and malt barley were processed into their respective flour prior to conducting the experiments as follows.

#### Maize grain flour production

The maize grain was hand sorted and cleaned. The grains were washed with tap water and rinsed in deionized water and finally soaked for 10 min (Mehra and Eckhoff 1996) and debranned using a traditional Ethiopian wooden pestle and mortar. Thereafter, the bran and grits were dried under the sun for easy separation and to reduce the moisture in the grits. Finally, the grits were grain milled (KARLKOLB D‐6072, Dreich, West Germany) and passed through the 0.5 mm sieve. The milled samples were packed in airtight sealed polythene bags and stored in a refrigerator for later use (about 5°C).

#### Barley grain malt flour production

The barley grains were cleaned manually and sieved to remove impurities and dockages. Malt was prepared under controlled steeping, germination, and kilning conditions as described in Galano et al. (2011). The cured sample was polished to remove the dried rootlets and acrospires and milled (KARLKOLB D‐6072, Dreich, West Germany) to pass through the 0.5 mm sieve. The flour was packed in airtight polythene bags and stored in a cool place (about 5°C) until used.

#### Pea grain flour production

Pea grain was cleaned, sorted, washed in tap water and rinsed with deionized water and soaked in water for two hours (Gibson and Hotz 2001). The soaking water was removed and rinsed with fresh water again and then sun dried. Finally, 100 g of the dried samples was roasted (350°C) on stainless steel iron pan, using an electric heating appliance according to the Ethiopian shiro processing method with some modification, that is, by reducing the roasting time to only 5 min. Then the roasted pea was milled (KARLKOLB D‐6072, Dreich, West Germany) to pass through the 0.5 mm sieve, packed in an airtight polythene bags and stored in a cool place (about 5°C) until used.

### Experimental design and treatment combinations

The experimental design was generated using d‐Optimal mixture design (Table 1) and the constrained region was indicated on Table 2. A mixture design is appropriate when the response depends on the component proportions of the mixture and not on the component quantities. The treatment consists of three mixtures: 55 < maize <90, 20 < pea <35, and 4 < malted barley <12 (maize + pea + malted barley = 100%). Accordingly, 14 samples as treatment combinations were generated.

**Table 1:** Ratios obtained by mixture design for the fourteen formulations of flour

Run	Maize (%)	Pea (%)	Barley (%)
1	67.5	24.3	8.2
2	55.0	35.0	10.0
3	61.0	35.0	4.0
4	61.0	35.0	4.0
5	76.0	20.0	4.0
6	68.0	20.0	12.0
7	68.5	27.5	4.0
8	59.0	31.3	9.0
9	63.0	28.6	8.4
10	76.0	20.0	4.0
11	72.0	20.0	8.0
12	61.0	26.5	12.0
13	55.0	35.0	10.0
14	68.0	20.0	12.0

**Table 2:** Constraint region of maize, pea, and barley taken by mixture design

Low	≤Constraint	≤High
0.55	≤A: Maize	≤0.95
0.20	≤B: Pea	≤0.35
0.04	≤C: Barley	≤0.12

A + B + C = 1.

### Proximate composition analysis

Nutritional composition of the experimental flour: moisture, crude fat, crude protein, crude fiber, and total ash were determined, using the AOAC Official Method Nos: 925.09, 4.5.01, 979.09, 962.09, and 923.03, respectively (AOAC 2000). The total carbohydrate was determined by FAO, 1998 was included in the reference list difference (FAO, 1998) based on the following formula. Results were expressed per 100 g of the flour.

Carbohydrate (%) = 100 ‐ (% Protein + % Moisture + % Crude fat + % Crude fiber + % Ash).

### Sensory analysis

Porridge was prepared from flour to water ratio (1:3), 10 g sugar was also added to all samples (Owino et al. 2007) and worked to uniform consistency for about five 5 min. The product was assessed for their color, texture, flavor, odor, and overall acceptability on 5‐ point hedonic scales.

### Statistical analysis

The three component constrained mixture design was conducted using Design‐Expert^®^ 6 (Stat‐Ease) Minneapolis MN, USA. Regression models, contour plot, and response surface were obtained using Minitab 16 statistical package. The significance test was set at *P* < 0.05. The fitted models for all parameters were generated in three‐dimensional contour plots. Graphical optimization was done to determine the optimum formulation point of maize, pea, barley, and carrot flour.

## Results and Discussion

### Proximate composition of the complementary foods

The moisture content, crude protein, crude fiber, crude fat, ash, and carbohydrate content of the complementary food flour formulations ranged between 5.0–6.6%, 13.0–18.5%, 3.06–4.45%, 1.8–2.5%, 1.5–2.5%, and 68.9–74.1% respectively (Table 3). Except for fat, the lack‐of‐fit was not significantly different (*P* < 0.05) (Table 4).

**Table 3:** Proximate composition and energy contents of the 14 different complementary food flour formulations

Ingredients in a mixture (%)				Proximate composition						
Run	M	P	B	MO %	CP%	CF%	Fat%	Ash%	CHO%	Energy (kcal/100 g)
1	67.5	24.3	8.2	5.8	14.0	4.1	2.4	1.9	72.2	366.4
2	55.0	35.0	10.0	5.0	18.0	3.5	1.8	2.3	69.5	366.2
3	61.0	35.0	4.0	5.4	17.00	3.26	2.1	2.5	69.16	365.15
4	61.0	35.0	4.0	5.4	17.05	3.21	2.1	2.5	69.10	365.11
5	76.0	20.0	4.0	6.4	13.0	3.4	2.5	1.6	73.2	367.3
6	68.0	20.0	12.0	5.6	13.8	4.0	2.2	1.5	74.1	371.0
7	68.5	27.5	4.0	5.9	15.0	3.8	2.2	2.5	71.2	364.4
8	59.0	31.3	9.7	5.2	16.0	3.4	2.0	2.4	71.1	366.4
9	63.0	28.6	8.4	5.5	15.5	3.1	2.1	2.0	72.4	370.5
10	76.0	20.0	4.0	6.5	13.0	3.3	2.5	1.6	73.4	368.2
11	72.0	20.0	8.0	6.0	13.5	3.7	2.5	1.5	73.1	368.4
12	61.5	26.5	12.0	5.5	14.5	3.8	2.1	1.5	72.6	367.5
13	55.0	35.0	10.0	5.0	18.5	3.3	1.9	2.4	68.9	366.7
14	68.0	20.0	12.0	5.6	13.8	4.0	2.2	2	72.7	365.6

M, maize; P, pea, B, barley; MO, moisture; CP, crude protein; CF, Crude fat; CHO, total carbohydrate.

**Table 4:** Predicted model for the proximate content analysis

Approximates	Model	*R* ^*2*^ value
Moisture	*Y = *7.9*X*_*1*_ *−* 5.79*X*_*2*_ * + * 26.58 *X*_*3 *_ *+ *11.76*X*_*1*_ *X*_*2 *_ *− *41.88 *X*_*1*_ *X*_*3 *_ *+* 1.95*X*_*2*_ *X*_*3*_	0.987
Crude protein	*Y = *16.4 *X*_*1 *_ *+ *106.9 *X*_*2*_ *−* 60.5 *X*_*3 *_ *− *138.5 *X*_*1*_ *X*_*2*_ * + *96.1 *X*_*1*_ *X*_*3*_ *−* 27.9*X*_*2*_ *X*_*3*_	0.988
Flavor	*Y = *4.06*X*_*1 *_ *+ *10.29*X*_*2 *_ *− *39.3*X*_*3 *_ *− *12.24*X*_*1*_ *X*_2 _ *+ *46.34 *X*_*1*_ *X*_*3 *_ *+ *59.55*X*_*2*_ *X*_*3*_	0.997
Overall acceptability	*Y = *4.12*X*_*1 *_ *+ *8.11*X*_*2 *_ *− *12.39*X*_*3 *_ *− *8.27*X*_*1*_ *X*_*2 *_ *+ *12.23 *X*_*1*_ *X*_*3 *_ *+ *40.01*X*_*2*_ *X*_*3*_	0.958

*X*_1_^ ^= maize, *X*_2_ = Pea and *X*_3_ = Barley.

### Moisture content

The moisture content for the 14 treatments of complementary food samples was ranged between 5.0 and 6.6%. The highest moisture content was recorded in blend of 76% maize, 20% pea and 4% malted barley while the least moisture content was observed in blend of 55% maize, 35% pea and 10% malted barley (Table 3). Moisture content was increased when the proportion of maize increased and roasted pea decreased in the blend. Our finding is in the range of Nelson (1992) who reported 3–8% of moisture content as a quality factor for the food prepared from cereals. It is also very close to that of Amankwah et al. (2009) report (5.0–6.0%) who formulated complementary foods from fermented maize, rice, soybean and fishmeal. However, the result is below the Ahmad et al. (2008) report (7.2–8.7%) who formulated complementary food from wheat and soybean flour. However, our result is slightly greater than the recommended moisture content (<5%) by CODEX CAC/GL 08. 1991) except in the blend of 55% maize, 35% pea and 10% malted barley flour. This may be because of steeping and drying methods employed during the preparation of flour (Gausman et al. 1951).

### Crude protein content

The protein content for the 14 treatments of complementary food samples had ranged from 13.0–18.5%. The highest protein content was recorded in blend of 55% maize, 35% pea, and 10% malted barley and the least was found in 76% maize, 24% pea, and 4% malted barley blend. Protein content was increased with increasing of pea and decreased with increasing of maize proportion in the blend (Table 3). The result is similar with Saeeda et al. (2009) who reported 14.4% of crude protein from rice, mong, potato and poppy seeds blend; 17.5% crude protein from wheat, pea, cabbage and groundnut blends, and 14.5% of crude protein from maize, lentil, carrot, and sesame blend. But, the result is less than that of Ijarotimi and Keshinro (2012) report (23.85–28.84%) who formulated from germinated and fermented popcorn but, higher than that of Bojňanská et al. (2012) report (12.57–16.28%) who formulated from wheat, lentil and chickpea flour (10–50%). The difference could be due to the difference in blending ratio and crop types used during the formulation. According to Gibson and Hotz (2001), blending of cereal‐based foods and their processing methods can improve the protein content of the flour. The required daily allowance for protein contents in the complementary foods is ≥15% (WHO/ FAO 2004). However, in our finding, this result can be satisfied when the proportion of pea in the blend is greater than 27%. This suggests that increasing of the pea can improve the protein content of complementary processed foods (Barac et al. 2010).

### Crude fat

The fat content for the 14 treatments of complementary food samples had ranged from 1.8 to 2.51%. The highest fat content was recorded in the blend of 76% maize, 20% pea and 4% malted barley and the least was found in 55% maize, 35% pea and 10% malted barley blend ratios. The crude fat content was increased with the increasing of maize and decreased with the increasing of malted barley proportion in the blend (Table 3). The result is similar with that of Ghavidel and Davoodi (2011) who reported 1.36% crude fat in complementary food processed from Wheat and Green gram, and 1.27% crude fat from Wheat and Lentil seed flour composites. However, the result is lower than that of Solomon (2005) report (11.5–24.8%) who processed complementary food from rice, maize, acha grains, soya beans, bambara nut, benniseed, carrot, garden egg, and crayfish. In addition, our result is less than the daily recommended fat content in complementary foods range from 10 to 25% (WHO/ FAO 2004). This is may be due to the differences in crop types used and the processing methods employed during the formulation of the flour. For instance, the processing methods (removal of outer part of maize and germ) have significant effect on reduction in fat content (Arif et al. 2011). In case of our finding, increasing the maize content can improve the fat content of complementary processed food.

### Crude fiber

The crude fiber content in the complementary food processed was ranged from 3.1–4.1%. The high crude fiber content was recorded in the blended ratio of 67.5% maize, 34.3% pea and 8.2% malted barley while the least fiber content was recorded in 63% maize, 28% pea and 8.4% malted barley blended ratio (Table 3). This is similar with the investigations of Hussain et al. (2012) who reported fiber content from 1.36–1.81% in a formulation of wheat and lentil‐based complementary food flour. The daily recommended allowance of crude fiber in the complementary food is <5%. Thus, all the complementary foods processed in this study meet this requirement.

### Total ash

The total ash content in the complementary foods processed was ranged in between 1.5% and 2.5%. The highest ash content was recorded in the blended ratio of 61% maize, 35% pea and 4% malted barley and least ash content was observed in 72% maize, 20% pea and 8% malted barley blends respectively (Table 3). The ash content has increased with increasing the proportion of pea in the blend. Similar results were reported by Bojňanská et al. (2012) and Gibson and Hotz (2001) when legumes (lentil and chick pea) are included into cereal‐based complementary foods. All the complementary foods processed in this study meets the recommended ash content by WHO/FAO (2004) in the complementary food (<5 g/100 g).

### Carbohydrates

The carbohydrate contents in the complementary food processed had ranged from 68.9–74.1%. The highest carbohydrate content was observed in the blended ratio of 68% maize, 20% pea and 12% malted barley. The least carbohydrate content was seen in the blended ratio of 55% maize, 35% pea and 10% malted barley (Table 3). With the increasing of maize content in the blend, the carbohydrate content was found to be increased. Processing method like dehusking may increases carbohydrates content in the diet (Abiodun and Adepeju 2011). Similar results were reported by Elemo et al. (2011) and Hussain et al. (2012) who reported 63.7%–77.4% of carbohydrate content in complementary food processed from sorghum and cowpea, and 61.24–70.73% from wheat and lentil composite flour, respectively. All the complementary foods processed in this work meet the carbohydrate content recommended by WHO/ FAO (2004) in the complementary food (≥65 g/100 g).

### Energy

The energy (caloric) content of the 14 complementary foods processed was ranged between 364.4–371.0 kcal/100 g. The highest energy content was observed in 68.5% maize, 27.5% roasted pea and 4% malted barley blended ratio and the least energy content was observed in the blend of 68% maize, 20% roasted pea and 12% malted barley (Table 3). Energy content increased with the increasing of malted barley in the complementary processed food. Similar result (376.23–376.27 kcal/100 g) was reported by Hussain et al. (2012) who formulated complementary food from wheat and lentil composite flour. However, the result is less than the recommended energy content by WHO/ FAO (2004) in the complementary foods (400–425 kcal/100 g). This may be due to the less fat content of the raw materials used in the formulation of the food.

### Sensory evaluations

Sensory attributes of the blended flour products were assessed by 50 untrained panelists. Of these, 18 were mothers and females of reproductive age of Jimma University. The score values were found in between range of 3.51–4.10, 3.59–4.20, 3.69–4.30, 3.00–4.20 and 3.68–4.25 for odor, flavor, texture, color, and overall acceptance of the porridge, respectively (Table 5). lack‐of‐fit for all sensory attributes were not significant (*P* ≥ 0.05) and the model is predictable.

**Table 5:** Sensory analysis of the 14 different complementary food flour porridges

Run	Ingredients in a mixture (%)			Sensory evaluation result on 5‐point hedonic scale				
	M	P	B	Texture	Odor	Color	Flavor	Overall acceptability
1	67.50	24.30	8.20	3.80	3.80	3.76	3.77	3.83
2	55.00	35.00	10.00	4.20	4.00	4.20	4.20	4.45
3	61.00	35.00	4.00	3.60	3.80	4.05	3.86	3.94
4	61.00	35.00	4.00	3.59	3.80	4.00	3.85	3.93
5	76.00	20.00	4.00	3.00	3.51	3.69	3.59	3.68
6	68.00	20.00	12.00	3.70	3.86	3.75	3.65	3.80
7	68.50	27.50	4.00	3.50	3.66	3.92	3.68	3.82
8	59.00	31.30	9.70	4.00	3.89	4.02	4.00	4.19
9	63.00	28.60	8.40	3.58	3.85	3.94	3.87	3.98
10	76.00	20.00	4.00	3.00	3.51	3.70	3.60	3.69
11	72.00	20.00	8.00	3.50	3.76	3.65	3.70	3.75
12	61.50	26.50	12.00	3.75	3.82	3.9	3.82	3.98
13	55.00	35.00	10.00	4.00	4.10	4.30	4.18	4.25
14	68.00	20.00	12.00	3.72	3.87	3.67	3.66	3.77

M, maize; P, pea, B, barley.

#### Odor

The sensory score of odor for the 14 treatments of complementary food samples had ranged from 3.51 to 4.10. The highest score was recorded in the blending ration of 55% maize, 35% roasted pea and 10% malted barley, and the least score was found in 76% maize, 20% roasted pea, and 4% malted barley (Table 5).

#### Flavor

The flavor acceptance of complementary processed food was found in between 3.58 and 4.20%. The highest flavor acceptance was scored in 55% maize, 35% roasted pea, and 10% malted barley composite and the least was scored in 76% maize, 20% roasted pea, and 4% malted barley blended ratio (Table 5). The flavor acceptance was increased when the proportion of pea was increased and maize was decreased. These could be because of release of flavor compounds by nonenzymatic (Maillard and caramelization) reactions during roasting of pea (Muhimbula et al. 2011). The process enhances the acceptability of porridge made from roasted flour, because of dextrinization, and starch breakdown (Mensah and Tomkins 2003). Germination is also known to enhance flavor acceptability of food products (Inyang and Zakari 2008).

#### Color

The color acceptance of complementary processed food in this study was found in range of 3.65–4.30%. The highest color acceptance was recorded in 55% maize, 35% roasted pea, and 10% malted while the least color acceptance was recorded in 70% maize, 20% roasted pea, and 8% malted barley blended ratio (Table 5). Color acceptance of the porridge was increased with the increasing of proportion of roasted pea in the composite flour. Similar results were reported by Pelembe et al. (2002) who studied sorghum– cowpea instant porridge by extrusion cooking.

#### Texture

The texture acceptance of the porridge of complementary processed food was found in the range of 3.00–4.20%. The highest texture acceptance was recorded in the composite flour of 55% maize, 35% roasted pea and 10% malted barley blended ratio and the least was recorded in 76% maize, 20% roasted pea, and 4% malted barley blended ratio (Table 5). The highest texture acceptance was recorded when the proportion of malted barley and roasted pea increased. This may be because of roasting and malting effects in the processing of pea and barley. According to Shim and Lim (2013), cooking and mixing cereals with legumes increases the porridge texture.

### Overall acceptability

**Figure 1:**
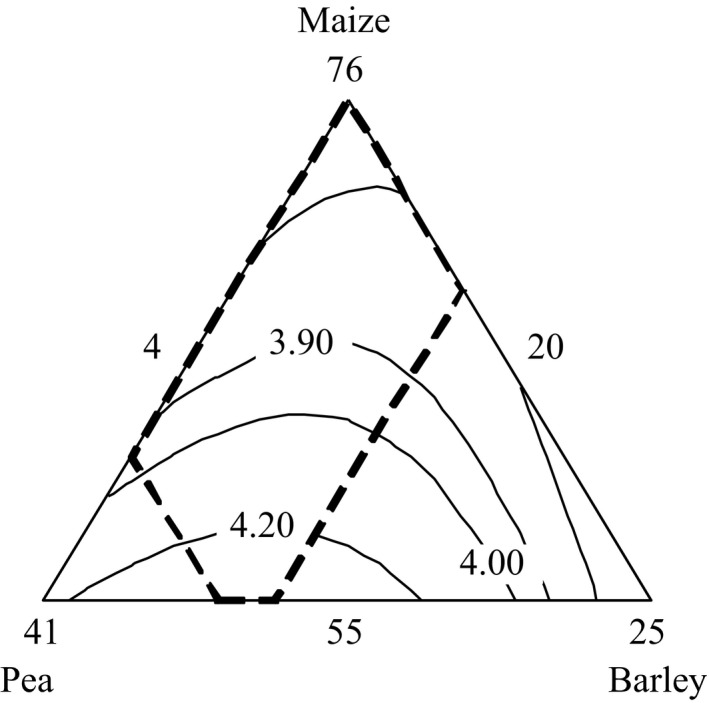
Mixture contour plot of overall acceptance.

The overall acceptability of the 14 treatments of complementary food samples had ranged from 3.68 to 4.45. The highest score had recorded in the proportion of 55% maize, 35% roasted pea, and 10% malted barley blended ratio and the least score was observed in 76% maize, % roasted pea, and 4% malted barley blended ratio (Table 5). The overall sensory acceptability of the porridge increased with the increasing of roasted pea (Fig.1). This result was in agreement with the investigations of Mbata et al. (2009) who reported high overall acceptability of complementary processed food formulated from maize–bambara ground nut. Compaore et al. (2011) also stated that the porridge from composite flour is organoleptically acceptable by the panelists in addition to its nutrient improvement.

### Superimposition of proximate composition and overall acceptability

**Figure 2:**
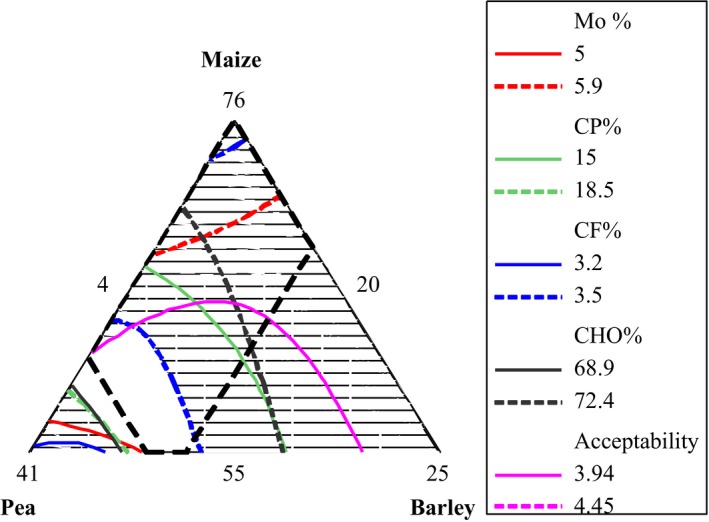
Overlaid contour plot of moisture content, protein, crude fiber, carbohydrate, and overall acceptability.

This study focused on determining the optimal formulation ratio of individual food source that can be suited to produce flour with desirable nutrient compositions. In order to determine the optimum formulation, the regions of acceptability in the contour plot for moisture content, protein, crude fiber, carbohydrate, and overall acceptability sensory attribute were superimposed looking at the daily recommendation of WHO/FAO (2004) for complementary foods. The sweet point of complementary food blended in this study (5.0–5.9% for moisture content, 15.0–17.5% for protein, 3.2–4.1% for fiber, 68.9–72.4% for Carbohydrate, and 3.94–4.45 for overall acceptability) was found in the flour samples prepared within the range of 55.0–68.5% for maize, 27.5–35.0% for roasted pea, and 4–10% for malted barley, respectively (Fig.**** 2).

## Conclusions

Based on our finding, the total moisture content, crude protein, crude fiber, crude fat, ash and carbohydrate of the complementary food flour had ranged between 5.3–5.7%, 13–18.5%, 3.06–4.45%, 1.8–2.5%, 1.5–2.5% and 68.9–74.1%, respectively. The sensory acceptability was found in between 3.51–4.10, 3.59–4.20, 3.69–4.30, 3.00–4.20, and 3.68–4.25 for odor, flavor, texture, color and overall acceptance of the porridge. In general, the optimum nutrient quality of the complementary food prepared from the flour of maize, pea, and malted barley was found to be in the range of 55.0–68.5% maize, 27.5–35.0% pea, and 4–10% malt barley. Such blending would lead to the complementary food contents to have 5.0–5.9% moisture content, 15.0–17.5% crude protein, 3.2–4.1% fiber, 68.9–72.4% carbohydrate content, and 3.94–4.45 of the overall acceptability.

## Conflict of Interest

None declared.
